# Acute Corneal Hydrops in Children with Primary Infantile Glaucoma: A Report of 31 Cases over 23 Years at the LVPEI

**DOI:** 10.1371/journal.pone.0156108

**Published:** 2016-06-01

**Authors:** Anil K. Mandal

**Affiliations:** Jasti V Ramanamma Children’s Eye Care Centre, L V Prasad Eye Institute, Hyderabad, India; National Eye Institute, UNITED STATES

## Abstract

**Purpose:**

Relatively little data exist regarding the outcomes of children with primary infantile glaucoma presenting with acute corneal hydrops. The aim of our study was to determine the surgical outcome of children of infantile glaucoma who presented with acute corneal hydrops.

**Methods:**

In total, 38 eyes of 31 consecutive children of infantile glaucoma presented with acute corneal hydrops who underwent primary combined trabeculotomy-trabeculectomy (CTT) by a single surgeon from January 1990 to December 2012 at the LV Prasad Eye Institute (LVPEI), a tertiary eye care centre in Southern India were enrolled in this retrospective study. Primary outcome measures were intraocular pressure (IOP) control (IOP ≤ 16 mmHg under anaesthesia or IOP ≤ 21 mmHg without anaesthesia) and clearance of corneal edema. Secondary outcome measures were visual acuity (VA), corneal diameter, bleb appearance, intraoperative and postoperative complications.

**Results:**

Mean age at presentation was 6.4 months (range, 2–11 months) and seven eyes (23%) had bilateral affliction. At presentation, all eyes (100%) had moderate to severe degree of corneal edema with a mean preoperative IOP of 25.6 ±5.1 mmHg. Postoperatively, the IOP reduced to 12.0 ± 3.8 mmHg (difference = -13.6, 95% CI = -15.7 to -11.5, t = -13.18, p<0.0001), and the percentage reduction in IOP was 53.05%. Preoperatively 83% of the eyes were on antiglaucoma medication, and postoperatively 2 eyes (5.3%) required 1 antiglaucoma medication for control of IOP. Preoperatively, corneal edema was present in all eyes and postoperatively it cleared in all of them. Significant myopic astigmatism was present in 28 eyes (74%), the commonest being compound myopic astigmatism (75%) followed by simple myopic astigmatism (21%). Normal VA (best-corrected VA; BCVA ≥ 20/60) was achieved in 44.4% of the eyes and 22.2% eyes had low vision (BCVA, <20/60 to 20/400). Complete success (IOP control and clearance of corneal oedema) was obtained in 94.7% eyes. There were no significant intraoperative or postoperative complications. Two thirds of the patients showed low, elevated functional filtering bleb. No patient had any bleb leak, blebitis or bleb related endophthalmitis. The median follow-up was 36 months (range 2–228 months).

**Conclusions:**

Primary CTT is safe and effective in controlling IOP, resulting in complete clearance of corneal edema with modest visual improvement in children of infantile glaucoma presenting with acute corneal hydrops. The outcome of the study will have a positive impact on counseling the parents preoperatively.

## Introduction

Infantile glaucoma is a form of developmental glaucoma that manifest after one month of birth until 3 years of age.[1] It manifests with megalocornea associated with increase in intraocular pressure (IOP) but it may remain undetected as the parents believe that the megalocornea may be a manifestation of “beautiful eye”. Consequently, parents of such children may not seek needful attention for medical advice. However some of the children with infantile glaucoma may go onto develop sudden corneal clouding because of acute corneal hydrops.[2–4] This is a medical emergency that prompts the parents to seek consultation with an ophthalmologist or a pediatrician. A detailed ophthalmic evaluation is mandatory to rule out the other causes of acute corneal hydrops.

Once the diagnosis is confirmed, early surgical intervention is required to control the IOP. Although there are various surgical options available in the armamentarium of glaucoma surgeons, goniotomy is technically difficult because of corneal edema.[5–7] External trabeculotomy is a suitable alternative as it is possible to perform this procedure even with severe degrees of corneal edema [8–11]; however, the long-term results of trabeculotomy are not encouraging in Indian patient population.[12, 13] Another surgical option in the form of primary combined trabeculotomy–trabeculectomy (CTT) is the best alternative as has been reported in various ethnic populations from different parts of the world.[14–18] Encouraged by our results of primary CTT in other forms of developmental glaucoma, we performed the same surgical procedure in infantile glaucoma.[12, 19–21]

Although acute corneal hydrops is a well recognized but rare manifestation of infantile glaucoma, extensive literature search failed to reveal any series reporting its management outcome. Therefore, the aim of the present study was to report the surgical and visual outcomes of children with infantile glaucoma who presented with acute corneal hydrops at the L V Prasad Eye Institute (LVPEI) [22], a tertiary eye care centre, in Southern India over a period of 23 years i.e.: from January 1990 to December 2012.

## Patients and Methods

We reviewed the medical records of all 31 children (38 eyes) with acute corneal hydrops due to infantile glaucoma who underwent glaucoma surgery by 1 surgeon (AKM) at the LVPEI, Hyderabad, South India from January 1990 to December 2012. A detailed ocular examination was performed under anesthesia to establish the diagnosis of infantile glaucoma with acute corneal hydrops. The parameters documented during this examination included: corneal findings (horizontal corneal diameter using calipers, clarity, presence or absence of Haab’s striae and its location) and intraocular pressure (IOP) using Perkin’s hand-held tonometer, In addition, a detailed examination of the lacrimal sac and nasolacrimal duct was performed to rule out the possibility of sac infection. Upon completion of the examination under anesthesia (EUA), the findings were interpreted to establish the diagnosis and decision was taken for surgical intervention. Procedures such as refraction, gonioscopy and fundus evaluation were not possible preoperatively given the presence of corneal edema in all cases.

The following information was collected for each patient: age at presentation (in months), gender, age at surgery, pre-and postoperative (at last follow-up visit) corneal diameter and clarity, and diameter, pre-and postoperative IOP, visual acuity, refractive error, number of antiglaucoma medications at last visit, bleb characteristics, complications, if any. The primary outcome was surgical success as observed at the last follow-up visit. Complete success was clearance of corneal edema and IOP of ≤ 16 mmHg in patients examined under general anaesthesia or < 21 mmHg in patients who were old enough to be examined with the slit—lamp. However, refractive error, gonioanomaly, disc evaluation were not considered in the definition of success given that corneal oedema prevented their assessment in the preoperative period. Consequently, comparison of these parameters was not possible between preoperative and postoperative visits Likewise, visual acuity was not considered in the definition of surgical success. Qualified success was defined when such IOP was maintained with one antiglaucoma medication (AGM). Failure was defined when such IOP could not be achieved even with the addition of one AGM; persistent corneal edema; reduction of vision to no light perception, devastating complications; additional glaucoma procedures (including cyclodestructive procedures). Devastating complications included endophthalmitis, retinal detachment or chronic hypotony.

Primary CTT was the surgical technique performed in all cases. The methods undertaken for primary CTT have been described previously.[12, 13, 19–21] Briefly, the Schlemm’s canal was dissected under a partial thickness limbal-based triangular sclera flap and trabeculotomy ab externo was performed on the sides of the radial incision trabeculectomy was then performed in the usual manner. In cases of bilateral affliction, after completion of surgery on the first eye, the second eye was operated using a similar technique but with new a set instruments, drapes, gown, gloves etc., simulating a surgical procedure on a different patient.

## Ethics Statement

The study was approved by the Ethic Committee of LVPEI. All methods adhered to the tenets of the Declaration of Helsinki. All patient records/information was anonymized and de-identified prior to analysis. The individual in this manuscript has given written informed consent (as outlined in PLOS consent form) to publish these case details.

### Postoperative Regimen

All patients were treated with topical corticosteroids (Prednisolone acetate 1% 6 times/day tapered over 6 weeks), cycloplegic (cyclopentolate 1% 3 times a day for 3 weeks) and topical antibiotic (4 times/day for 1week). All patients were examined on 1^st^ postoperative day, 1 week, 3 weeks in the office followed by examination under anesthesia at 6^th^ week, and every 3 months for a year and then 6 monthly thereafter. At each visit, patients were underwent EUA during which a hand-held slit-lamp biomicroscope was used for an assessment of the anterior chamber depth corneal clarity and presence or absence of Haab’s striae, horizontal corneal diameter, IOP, bleb appearance, direct gonioscopy using Koeppe’s gonio lens and fundus evaluation including an assessment of the optic disc, using direct and indirect ophthalmoscope. Visual acuity (VA) was measured using age-appropriate methods. These included grating acuity assessment using Teller Acuity Cards [23] (TAC) (Vistech Consultants Inc., Dayton, OH, USA) for infants and toddlers and the grating acuity was, however, converted to Snellen equivalent for purposes of analysis and comparison based on the norms provided by Mayer et al. [24]. Other assessment procedures included Lea symbols [25] for preschoolers and the logMAR chart for school-aged children and adolescents. Retinoscopy was performed manually using a streak retinoscope (Heine, Beta 2000) under cycloplegia (1% cyclopentolate hydrochloride eye drops) in the postoperative period.

### Statistical Analysis

Descriptive statistics were employed to summarize demographic and outcome characteristics of the participants. Paired t test was used to compare the pre-and postoperative IOP. A P value less than 0.05 was considered statistically significant. All data analyses were performed using SPSS Statistics for Windows version 19.0 (IBM Corp. Armonk, NY).

## Results

A total of 31 patients (38 eyes) were identified from our retrospective chart review. The demographic characteristics of the participants are summarized in Table 1. The mean age at surgery was 6.4 months (range, 2–11 months). The majority had unilateral affliction (77%) while 7 (23%) had bilateral affliction. The mean follow-up was 54.4 ± 58.2 months (range, 2–228 months; median, 36 months). There was significant lowering of the mean IOP from preoperative (25.6 ± 5.1 mmHg) to the postoperative visit (12.0 ± 3.8 mmHg; difference = -13.6, 95% CI = -15.7 to -11.5, t = -13.18, p<0.0001), and this reduction was by a little over 50%. At the last visit, two eyes (5.3%) required one antiglaucoma medication (Timolol Maleate 0.5%) for control of IOP.

**Table 1:** Participant characteristics of 38 eyes of 31 patients with acute hydrops secondary to Infantile Glaucoma.

Demographic	No. (%)
Age at surgery	
Mean ± SD	6.4 ± 2.57
Range	2–11
Gender	
Male	16 (52)
Female	15 (48)
Affliction	
Unilateral	24 (77)
Bilateral	7 (23)
Horizontal corneal diameter at presentation (mm)	
Mean ± SD	13.2 ± 0.7
Range	11.5–14.5
Corneal clarity at presentation	
Edema	38 (100)
Corneal clarity at last visit	
Clear	38 (100)
Preoperative intraocular pressure (mmHg)	
Mean ± SD	25.6 ± 5.1
Range	16–36
Postoperative intraocular pressure (mmHg)*	
Mean ± SD	12.0 ± 3.8
Range	8–15
Reduction in intraocular pressure (mean, %)	53.05
Follow-up (months)	
Mean ± SD	54.4 ± 58.2
Range	2–228
Median	36

SD- Standard deviation;

*intraocular pressure recorded at last follow-up visit, P<0.0001.

### Corneal diameter and clarity

**Fig 1:**
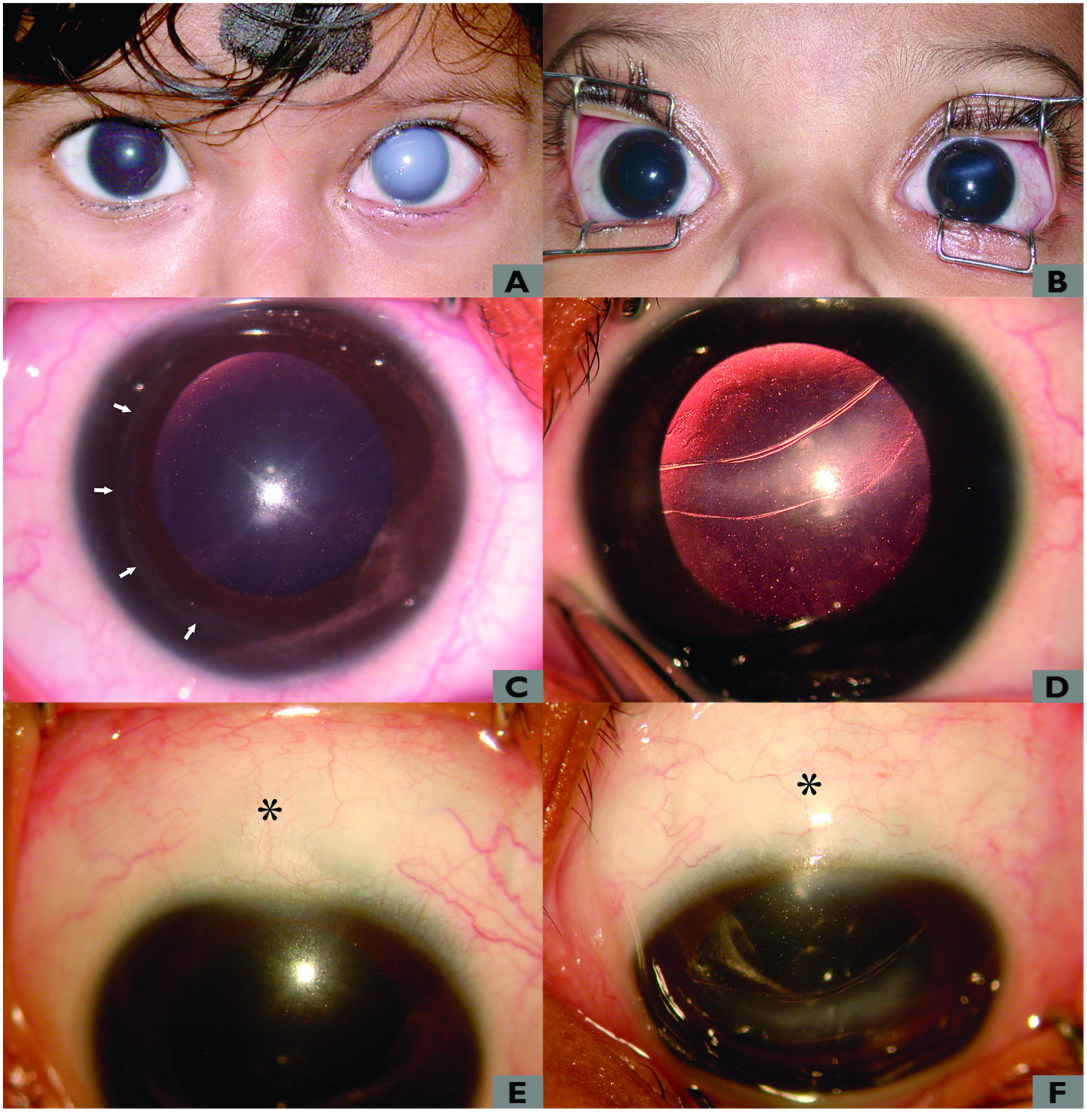
Clinical photograph of a child with bilateral acute corneal hydrops secondary to infantile glaucoma. A) Preoperative appearance showing corneal edema which is more severe in the left eye. B) One-year postoperative appearance of the child seen in figure A showing clear cornea in the right eye with linear horizontal corneal scar and Haab’s striae in the left eye. C) Photograph of the right eye in higher magnification showing Haab’s striae as curvilinear parallel lines in the temporal side (white arrows). D) Fundus retroillumination picture of the left eye showing Haab’s striae as illuminated parallel curved lines crossing the pupillary area. E) Clinical photograph of the right eye showing bleb appearance after 1 year of surgery (asterisk). F) Clinical photograph of the left eye showing the bleb appearance after 1 year of surgery (asterisk).

Mean preoperative horizontal corneal diameter was 13.2 ± 0.7mm (Range 11.5–14.5 mm). Although preoperative corneal edema was present in all the eyes, it cleared in all of them (100%) postoperatively. Fig 1A and 1B show the preoperative and 1-year postoperative appearance of both the eyes of a child who underwent simultaneous bilateral CTT at the age of 6 months. Similarly, Fig 1C and 1D show the preoperative and 1-year postoperative appearance of the right and left cornea with Haab’s striae of the same child. Fig 1E and 1F show the 1-year postoperative bleb appearance of the right and left eyes respectively.

### Refractive error

Data about refractive error was available for all eyes. One eye was emmetropic, 9 eyes had simple myopia and 28 eyes (74%) had astigmatism—the commonest being compound myopic astigmatism (75%) followed by simple myopic astigmatism (21%) and mixed astigmatism (4%).

### Visual acuity

At the final post-operative visit, best-corrected visual acuity (BCVA) was available for 27 eyes. Of these, 12 eyes (44.4%) had BCVA ≥ 20/60 and 6 had (22.2%) had BCVA ranging from 20/80 to 20/400. Nine eyes (33.3%) had BCVA less than 20/400.

### Success rate

Complete success as defined in the present study was obtained in 36 eyes (94.7%). Two eyes (5.3%) were classified as failures.

### Surgical complications

Intraoperative hyphema occurred in 1 eye and was well managed. Postoperative persistent shallow anterior chamber was noted in 1 eye which was surgically reformed with air bubble injection into the anterior chamber on the third postoperative day.

### Repeat surgery

Repeat trabeculectomy with mitomycin-C (MMC) was performed in one eye for uncontrolled IOP despite 3 AGMs. Trans-scleral cyclophotocoagulation was done in one eye of a patient with advanced disc damage and poor visual potential.

### Post-operative complications

No patient had any blebitis, bleb leak or bleb-related endophthalmitis.

## Discussion

Our study evaluated the results of primary combined CTT in infantile glaucoma presenting with acute corneal hydrops. We found good IOP control (≤16 mmHg) without AGM in 95% eyes and normal corneal clarity was achieved in all eyes. The results of visual outcome in our series are comparable to that reported by other investigators in the literature.[26, 27] Nonetheless, visual outcome was sub-optimal in little over 50% of the eyes, with a final best-corrected visual acuity of <20/60 in our study. Such a visual outcome is the consequence of high corneal astigmatism (up to 7 diopters of cylinder) from Haab’s striae running across the papillary area in most of the eyes.

The development of acute hydrops in our patient series is because of breaks in the descemet’s membrane under the influence of high IOP in an eye with pre-existing megalocornea. The aqueous humor gains access into the corneal stroma leading to sudden corneal edema.[2] This sudden corneal clouding prompted the parents of children with infantile glaucoma to seek urgent medical attention and consequently early glaucoma surgery.[3] We believe that in the absence of acute hydrops these children with silent infantile glaucoma would have remained undetected and it is plausible that further glaucomatous damage may have occurred.

Mean age at presentation of our patients was 6.4 months (range, 2–11 months) and most of the initial visual development is complete within the first 6 months of life.[28, 29] Naturally one would have expected excellent visual outcome in these children following CTT. However, we did not obtain such excellent results in the present series. We offer a couple of reasons for this. Firstly, the Haab’s striae seen in these children crossed the papillary area in most of the cases resulting in photophobia and high irregular corneal astigmatism, and this has the potential to produce meridional amblyopia. Secondly, there were corneal scars resulting from Haab’s striae—a permanent sequel and this may act as an impediment to good visual recovery.[30] In the present series, three-quarters of the eyes had high compound myopic astigmatism with cylindrical component as high as 7 diopters and despite aggressive amblyopia therapy, the visual outcome remained sub-optimal in about 50% of the eyes. Pragmatically, prompt surgical intervention at an early age as in our series should have reduced the risk of deprivation amblyopia, but our data have not borne this out. Similar results of amblyogenic factors resulting in visual impairment have been reported in the literature. [31] Nonetheless, the results of visual outcome in our series should be interpreted with caution given that there is also a concurrent maturation of the visual system with increasing age of the child that may have contributed to the improvement of VA and changed the refractive status.

Primary CTT is a promising surgical technique for the management of different forms of developmental glaucoma in the Indian context.[12, 13, 19–21] The alternative surgical technique of goniotomy is technically impossible given the presence of significant corneal edema from acute hydrops.[12, 20, 21] In the present series significant corneal edema was present in all the eyes and trabeculotomy abexterno may have been a viable option, but long term results of such a procedure is not encouraging in our patient population.[12] Primary CTT offers advantages of the dual mechanism of IOP control (trabeculectomy and trabecultomy) and good long term results have been reported by us previously in various forms of developmental glaucoma. [12, 13, 19–21] In our series, 84% of the eyes were using AGM at the time of presentation to us. By comparison, only 2 eyes (5%) required only a single AGM postoperatively. Two-thirds of the eyes showed mild to moderate filtering bleb which was low to moderately elevated with microcystic changes. These blebs are not prone for infection and we did not have any bleb-related infection or endophthalmitis in the present series.

Although a majority of our patients had unilateral acute corneal hydrops, 7 children (23%) had bilateral affliction, albeit with asymmetric severity. We performed simultaneous bilateral CTT in these children given our previous experience with such a procedure.[32] This resulted in early visual rehabilitation, reduced costs and reduced hospital stay and these augur well for the children and their parents.[32] However extreme care should be taken to prevent infection and the second eye should be operated using a new set of instruments and preparing the second eye simulating the surgical procedure on a different patient.[32]

Children with infantile glaucoma presenting with acute corneal hydrops require prompt surgical intervention. It has been established in the literature that the success rates are highest with the first surgical procedure.[6, 11, 18, 33] Furthermore, there is always a search for the best surgical technique that should be employed in a given patient.[34–37] Recently, 360° trabeculotomy has emerged as an attractive choice which can be performed with 6’O Proline suture or with the help of an illuminated microcatheter.[17, 38–41] Several encouraging reports have emerged in the literature favoring circumferential trabeculotomy with illuminated microcatheter. [17, 39–41] Steep learning curve and the cost of therapy using disposable illuminated microcatheter is a limiting factor especially in our scenario of a developing country. In a comparative study from India illuminated microcatheter assisted circumferential trabeculotomy achieved comparable results to primary CTT with mitomycin-C.[17] In the present study, we performed CTT without antifibrotic therapy, and achieved excellent IOP control. However, the long-term results remain unknown.

In the present series, none of the patients required second antiglaucoma surgery. If needed, MMC-augmented trabeculectomy or glaucoma drainage device are viable options as second surgical procedures.[42–44] The benefits of good IOP control in the long term with a single surgical procedure as obtained in the present study are encouraging for the treating ophthalmologist and we believe that such results will have a positive impact while counseling the parents preoperatively. However, the risk of long term drifts in IOP control is well known [42, 43] and the life-long follow-up of these children is mandatory. It is also possible that perhaps in the future, with advances in corneal treatment methods, these patients may be able to undergo corneal treatment of their high astigmatism as well as Haab's striae after the IOPs have stabilized and corneal edema cleared to help rehabilitate their vision and prevent amblyopia.

Limitations of this study include the flaws inherent in a retrospective study including the nonrandomization of patients. This study analyzed the surgical experience of a single glaucoma surgeon. Moreover, the mean follow-up was 54.4 ± 58.2 months (range, 2–228 months; median, 36 months) so the longer-term outcomes of primary CTT in a similar patient population remain to be determined.

In conclusion, primary CTT showed promise as an initial surgical procedure in the management of children with infantile glaucoma with acute corneal hydrops. The IOP control is excellent with acceptable bleb appearance with no bleb-related complications and moderate visual recovery in our patient population. Hence, same surgical procedure may be a viable option in other ethnic populations.
